# Advancing environmental epidemiologic methods to confront the cancer burden

**DOI:** 10.1093/aje/kwae175

**Published:** 2024-07-20

**Authors:** Rebecca D Kehm, Susan E Lloyd, Kimberly R Burke, Mary Beth Terry

**Affiliations:** Department of Epidemiology, Mailman School of Public Health, Columbia University, New York, NY 10032, United States; Department of Epidemiology, Mailman School of Public Health, Columbia University, New York, NY 10032, United States; Herbert Irving Comprehensive Cancer Center, Columbia University, New York, NY 10032, United States; Department of Epidemiology, Mailman School of Public Health, Columbia University, New York, NY 10032, United States; Herbert Irving Comprehensive Cancer Center, Columbia University, New York, NY 10032, United States; Silent Spring Institute, 320 Nevada Street, Suite 302, Newton MA 02460, United States

**Keywords:** environmental carcinogens, cancer, environmental epidemiology, study design, genetic susceptibility

## Abstract

Even though many environmental carcinogens have been identified, studying their effects on specific cancers has been challenging in nonoccupational settings, where exposures may be chronic but at lower levels. Although exposure measurement methods have improved considerably, along with key opportunities to integrate multi-omic platforms, there remain challenges that need to be considered, particularly around the design of studies. Cancer studies typically exclude individuals with prior cancers and start recruitment in midlife. This translates into a failure to capture individuals who may have been most susceptible because of both germline susceptibility and higher early-life exposures that lead to premature mortality from cancer and/or other environmentally caused diseases like lung diseases. Using the example of breast cancer, we demonstrate how integration of susceptibility, both for cancer risk and for exposure windows, may provide a more complete picture regarding the harm of many different environmental exposures. Choice of study design is critical to examining the effects of environmental exposures, and it will not be enough to just rely on the availability of existing cohorts and samples within these cohorts. In contrast, new, diverse, early-onset case-control studies may provide many benefits to understanding the impact of environmental exposures on cancer risk and mortality.

**This article is part of a Special Collection on Environmental Epidemiology**.

## Introduction

Environmental exposures may contribute to carcinogenesis through mechanisms such as genomic and epigenetic alterations, endocrine disruption, and adipogenesis.^1^^‑^^3^ The International Agency for Research on Cancer has classified 126 agents as carcinogenic to humans (Group 1), 95 agents as probably carcinogenic to humans (Group 2A), and 323 agents as possibly carcinogenic to humans (Group 2B).^4^ The key distinction between Group 1 and Group 2 classifications is whether there is sufficient (Group 1) or limited (Group 2) evidence in humans.^5^ With few exceptions (eg, medications in randomized clinical trials), most Group 1 agents have been identified through observational epidemiologic studies conducted in highly exposed populations such as occupational cohorts. Thus, epidemiology is at the heart of determining sufficiency of carcinogenicity evidence.

Epidemiologic evidence also is crucial for state and national cancer control policies, especially given that population attributable risk (PAR) estimates are sometimes used by policymakers to inform state cancer control plans.^6^ Yet, PARs may provide an incomplete picture for individual risk factors given that cancers are multifactorial in etiology. For example, while PARs for both environmental agents and occupational exposures have been in the 3%-5% range of all cancers combined,^7^^‑^^10^ with little change in these estimates over the past half century,^8^^‑^^10^ it has been estimated that a much higher percentage of specific cancers result from environmental exposures (eg, 80% of mesotheliomas are attributable to environmental exposures^11^^,^^12^). Further, the precision of causal attribution estimates depends on the accuracy of exposure measurement. Therefore, PARs are likely to be more accurate for constructs that are easy to measure, such as smoking, which is easy to recall and has been validly measured for well over half a century.^13^^,^^14^ By contrast, many environmental exposures are difficult to measure because they are ubiquitous, can be harmful even at low doses of exposure, and are often correlated with other exposures such as smoking status.^15^ Given that cancer rates are increasing in younger cohorts (adults aged <55 years)^16^ despite that smoking rates are declining,^17^^‑^^19^ it may now be easier to detect the effects of environmental exposures that were harder to measure in the past because of their correlation with smoking. Yet, improved study designs and measurement techniques may also be needed to advance the field of environmental epidemiology.

Epidemiologic advancements in the study of environmental carcinogens are also essential for addressing cancer disparities. Racial and ethnic minoritized and socioeconomically disadvantaged populations not only have higher exposure to environmental contaminants (eg, community and household chemicals/pollutants,^20^^‑^^22^ personal care and dietary products^23^^‑^^26^) but also frequently experience heightened vulnerability to the deleterious health effects of environmental toxicants.^27^^,^^28^ Yet, most cancer cohorts do not adequately represent these populations. As a result, we have been unable to discern the extent and nature of joint/interactive contributions of environmental and social factors in producing and sustaining cancer inequities, as has been demonstrated for other health outcomes (eg, cardiometabolic conditions).^29^

In this article, we examine study design methods for advancing the human evidence on environmental carcinogens. We do this by (1) discussing improvements in exposure measurement, (2) examining the gains from integration with multi-omic measures, and (3) rethinking the hierarchy of study designs. We end with the case study of breast cancer (BC), the most common cancer in women globally,^30^ to demonstrate why choice of study design and timing of exposure measurement are critical for examining environmental carcinogens.

## Measurement of environmental exposure constructs

### Occupational contrasts and classifications

The connection between certain environmental exposures and cancers has been known for centuries, primarily through the observation of higher rates of certain types of cancer occurring in individuals in certain occupations as compared with the general population (eg, scrotal cancers among chimney sweeps^31^ and bone cancers among radium dye painters^32^^,^^33^). Different approaches have evolved over time to more accurately measure exposures in occupational settings with complex exposure mixtures (eg, see Siemiatycki et al^34^). Job exposure matrices remain an important method with which to classify individuals based on exposure for different industries, but their utility in the future will depend on whether matrices continue to be updated over time to reflect contemporary job categories.

### Self-reporting of environmental exposures

Outside of the occupational setting, it has been much more challenging to capture past environmental exposures, particularly for cancers with long induction times. Questionnaires have been used in many epidemiologic studies to query about past exposure (eg, see Nieuwenhuijsen^35^). Yet there remains substantial measurement heterogeneity because individual-level (eg, personal care products, food items, cooking methods), household-level (eg, cleaning products, furniture, indoor air pollution), and community-level (eg, water, outdoor air pollution) exposures may be differentially recalled. Further, given the ubiquity and low dose of many environmental exposures in the general population, it may be difficult for individuals to accurately self-report their exposure levels.

### Biomarkers from biospecimens

The field of molecular epidemiology^36^^,^^37^ has provided ways to measure exposures without relying on recall through questionnaires and/or job matrices. These include short-term exposure measurements in urine and longer-term exposures in blood or even toenails, teeth, or hair.^38^^‑^^44^ For example, short-term (previous 24-48 hours) exposure to polycyclic aromatic hydrocarbons (PAHs) from air pollution, active and passive smoking, certain cooking methods, and diet can be measured in urinary metabolites.^45^^,^^46^ Biomarker levels can be measured before and after treatment^47^ to examine whether the treatment itself affects the biomarker, but it is still possible that the underlying disease process may lead to reverse caution. This is particularly true if the disease leads to weight loss, as many chemicals may be stored in adipose tissue.^48^ In this case, prospective cohort studies and nested case-control studies will be essential for studying environmental exposures because these study designs allow for the collection of biomarker data prior to cancer development. Childhood cohorts with biomarker data available from early life may also be useful for nested case-control studies of cancers or, if underpowered for cancer, intermediate markers of cancers.

Biomarkers can also be used to measure the effect of environmental exposures on bodily systems that may lead to carcinogenesis. For example, tissue-based biomarkers in cancer research have been essential to understanding how environmental chemicals affect tissue-specific changes (eg, early work on environmental chemicals and p53 mutations^49^ now extended to somatic mutational signatures and environmental exposures^50^). Assessment of blood and tissue-based DNA adducts, as well as other assays of DNA damage, can also be used to measure the effects of environmental exposures on carcinogenic processes.^51^ Yet, there are limitations to using biomarker data to measure environmental exposures. Biospecimen collection can be challenging and cost-prohibitive (costs for sample collection, storage, and laboratory assays), especially in larger studies.^52^ Further, biomarkers may not always capture the relevant susceptibility period, especially for exposures with short biological half-lives.^53^

Historically, environmental epidemiologists have studied individual classes of chemicals and their health effects in isolation. However, growing appreciation for the fact that humans are exposed to many chemicals simultaneously, which may interact synergistically to affect health, has led to advanced methodologies for studying chemical mixtures.^54^ This includes the use of passive sampling devices, such as silicone wristbands to measure personal exposure to chemical mixtures.^55^^,^^56^ The growing number of new chemicals in the environment (the Chemical Abstract Service Registry grew from 20 million chemicals to 156 million between 2002 and 2019)^54^ has also signaled the need for more untargeted approaches to studying environmental exposures and health. This has led to the emerging concept of the “exposome,” which strives to encompass all exposures, including exposure to environmental chemicals, dietary constituents, psychosocial stressors, and physical factors, as well as their corresponding biological responses.^57^

### Database linkages

The ability to link to public databases of common environmental exposures has improved the measurement of prior exposures. This approach also overcomes some of the limitations of other data collection techniques (eg, the issue of recall bias when using self-reported data; issues of feasibility and expense when using biomarker data). Some examples of national databases include the Centers for Disease Control and Prevention’s National Environmental Public Health Tracking Network^58^ and the Environmental Protection Agency’s EJScreen^59^ (for more examples, refer to Table S1). Many state and local agencies also have developed databases to track environmental exposures of specific concern in their communities (eg, see the New York State Environmental Public Health Tracking Program^60^). These environmental databases can be used to develop cumulative exposure models for both community- and individual-level air pollution exposures. For example, machine learning algorithms were used to predict daily and annual concentrations of fine particles (particulate matter with an aerodynamic diameter ≤ 2.5 μm) as early as the year 2000.^61^^‑^^67^ In combination with the collection of residential history data, this allows for the characterization of prior air pollution exposures with 5-, 10-, 15-, and even 20-year lags prior to cancer diagnosis. Additionally, environmental databases can be linked to other community-level data sources to examine associations between environmental exposures and neighborhood characteristics, structural racism, and other social determinants of health to identify communities with increased susceptibility to environmental carcinogens (eg, see Shkembi et al^68^).

While database linkages have expanded the options for measuring environmental exposures, they are limited in their application. For instance, database linkages are not readily available for the measurement of individual- and household-level exposures to environmental carcinogens in personal care, cosmetic, and household products—studies of which still rely primarily on self-reporting. Consumer product purchasing databases could help to bridge this gap, but they often are cost-prohibitive to access. Further, community-level data do not capture interindividual variability in exposure levels. Therefore, while community-level data are essential for policy questions, this approach should be coupled with other data collection approaches (eg, biomarker data) in understanding disease etiology. Additional considerations arising when selecting these different measurement approaches are summarized in Table 1.

**Table 1:** Potential sources of bias and exposure timing considerations when using different environmental exposure measurement methods.

**Measurement method**	**Selection bias**	**Information bias**	**Confounding**	**Exposure timing**
Occupational classifications	Healthy worker effect limits external comparisons; internal dose response may be more valid.^69^^‑^^71^ May not always be generalizable to non–occupationally exposed individuals given dose and duration differences.^72^ Women are historically less likely than men to be included in occupational studies.^73^	Measurement error will depend on completeness of employment records and how well they capture cumulative exposures.^74^ Most likely to be nondifferential bias because self-reporting is usually not involved.^75^	Many industry records may not have data on other cancer risk factors (eg, smoking).^76^ Many industries have complex exposure mixtures, making it challenging to separate out effects.^77^ May be difficult to disentangle confounding by socioeconomic status, which is associated with job category.^78^	Occupational studies can be conducted retrospectively and so can test hypotheses regarding induction time and windows of susceptibility.^79^ It may be difficult to study people who change occupations frequently.^80^
Self-reporting by questionnaire	In case-control studies, depends on whether selection is based on self-reported exposure status.^81^ In cohort studies, depends on whether follow-up is different based on self-reported exposures at baseline.^81^	Reliability of recall will depend on: (1) the environmental exposure of interest, (2) the time in life queried about, and (3) whether the exposure occurs at the individual, household, or community level.^35^ May be differential bias in case-control studies.^82^	Depends on the quality and completeness of self-reported confounders.^83^ Key concern is whether errors in reporting of environmental exposures and confounders are related.^83^	Often questionnaires do not capture a complete history of environmental exposures prior to cancer. This is more feasible in case-control studies.^35^ Cohort studies may not have updated environmental exposures on a regular basis and may not ask about exposure history at baseline.
Biomarkers	Depends on whether there are differences between those who agree and disagree to biospecimen collection.^84^^,^^85^ It may be more of an issue for samples that are harder versus easier to collect (eg, tissue vs urine).^52^	Most likely to be nondifferential bias if laboratory is blinded to case status and samples are analyzed in a random order.^86^	Environmental exposures are often correlated,^87^ making it difficult to separate out effects. Advances in mixture modeling^88^ and exposomics^57^ are addressing this issue.	Biomarkers from urine and shorter-term markers in the blood only reflect exposure levels over the last few days or months, respectively. The use of these biomarkers in cancer studies thus assumes that exposures are steady across time.^53^^,^^89^ Tissue-based biomarkers and molecular signatures of exposures may be used as measures of effect.^90^^,^^91^
Database linkage	Less of a concern than other methods, if coverage is relatively complete.	Measurement error more likely for exposure assessment at the individual level if residential history is not complete and/or a poor proxy of exposure.^92^ May be differential bias in case-control studies if recall of residential histories influenced by case status.^82^	Depends on ability to link to other data sources, including questionnaires, electronic health records, and other large databases (eg, US Census Bureau). Less of an issue in ecological studies if inferences are made at the ecological level (eg, public policy).^93^	Increasingly state, federal, and even global databases go back several decades (see Table S1 for examples). May still not have enough data yet, depending on the age of diagnoses and the induction time hypothesized.

## The role of “-omics” in epidemiologic studies of environmental exposures

Advances in high-throughput technologies have led to a growing field of multi-omics platforms, or large biomarker datasets characterizing biological features, including genomics, epigenomics, transcriptomics, proteomics, and metabolomics.^94^^,^^95^ In cancer research, a key way of developing and understanding the role of environmental carcinogens may be through the integration of environmental exposures with these established and emerging multi-omic platforms. To date, genomics is the most widely utilized platform in epidemiologic studies; it has been used to identify thousands of genetic variants associated with different types of cancer in human populations, as well as to study gene–environment interactions. Increasingly, genomics data are used to conduct mendelian randomization studies, which use genetic variants as proxies for exposures to remove confounding.^96^ Although genomics can be measured at any time, studies that collect genomic samples in older individuals will very likely be biased by survivorship. The use of other multi-omic platforms also requires careful consideration about the timing of sample collection, given that these other endpoints can change over time and may operate as exposures, modifiers, or mediators depending on when they are measured (for details, see Table S2).

## Contextualizing the hierarchy of study designs

While randomized clinical trials are on top of the study design hierarchy in evidence-based medicine schema, most human carcinogens have been identified through observational designs. For at least 2 decades, cohort studies in cancer have been increasingly deployed around the United States and globally, particularly for diet studies, but also for many other exposures, including environmental exposures. There are many benefits to using cohort studies, including temporality and the ability to measure exposures without knowing the outcome.

### Who is missing from cancer cohort studies?

The challenge of cohort studies, however, is that they require many individuals followed over a long period of time in order to capture enough outcomes to be well-powered to detect significant associations. In many cancer cohort studies, the investigators typically start recruitment at age 40 years or later and end up having a wide range of recruitment ages (eg, 40-70 years) at baseline.^97^ Because high exposure to certain environmental agents might lead to premature mortality due to either cancer or other causes (eg, cardiovascular and respiratory diseases), this means that older population-based cohorts may in fact be missing people with the highest exposure levels. This also means that these cohorts may underrepresent communities of color and socioeconomically disadvantaged populations, given that these are the groups disproportionately burdened by environmental exposures and other adverse health conditions that can lead to premature mortality. Further, it can be challenging to measure early-life exposures in cohort studies that begin recruitment in midlife because biomarker data are often not available prior to study enrollment. This is an important limitation of many cohort studies, given that the latency of tumorigenesis suggests that early-life environmental exposures contribute to carcinogenesis.^98^

**Figure 1:**
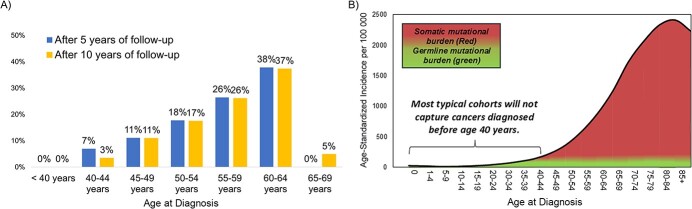
Theoretical age distribution (A) and mutational burden (B) of incident cancers in a cohort study with recruitment in midlife. The graph shows the age distribution of incident cancer cases in a hypothetical cohort with recruitment from age 40 years to age 64 years, assuming equal recruitment numbers for each age group (40-44, 45-49, 50-54, 55-59, and 60-64 years). Age-specific cancer incidence rates are estimated using age-specific cancer incidence data from the Centers for Disease Control and Prevention’s National Program of Cancer Registries and the Surveillance, Epidemiology, and End Results program (2001-2019 data representative of all 50 US states). A) Example of age distribution of incident cancer cases in a cohort with recruitment at ages 40-64 years; B) distribution of germline and somatic mutations by age group at diagnosis (adapted from Qing et al^99^). The germline variant burden in cancer genes correlates with age at diagnosis and somatic mutation burden.

Additionally, cohort studies that recruit individuals in midlife will largely miss incident cancers in younger adults. For example, in a cohort study with recruitment between ages 40 and 64 years (assuming equal distribution across this age range), it is estimated that less than 20% of incident cancers will occur in enrollees aged 40-50 years at diagnosis; none will occur in enrollees younger than age 40 years at diagnosis (Figure 1A). This means that most of the incident cancers in cohort studies with recruitment in midlife will be attributed to somatic, rather than germline, mutations. This is because the source of susceptibility is strongly correlated with age, such that the proportion of cancers due to somatic mutations increases with age (Figure 1B).^99^ The number of incident cancers occurring from germline mutations is further reduced by the fact that most cancer cohort studies exclude individuals with a history of cancer. This is a type of left-truncation bias that is sometimes described as “deleting the susceptibles,” as the proportion of cancers due to germline mutations is higher at younger ages.^99^ Somatic mutations can also increase susceptibility to carcinogens, but they can only be measured in tissue samples, which are often hard to collect. Therefore, it is difficult to leverage data on genetic susceptibility in existing cancer cohort studies with recruitment in midlife.

### Alternative cohort designs for studying environmental carcinogens

**Figure 2:**
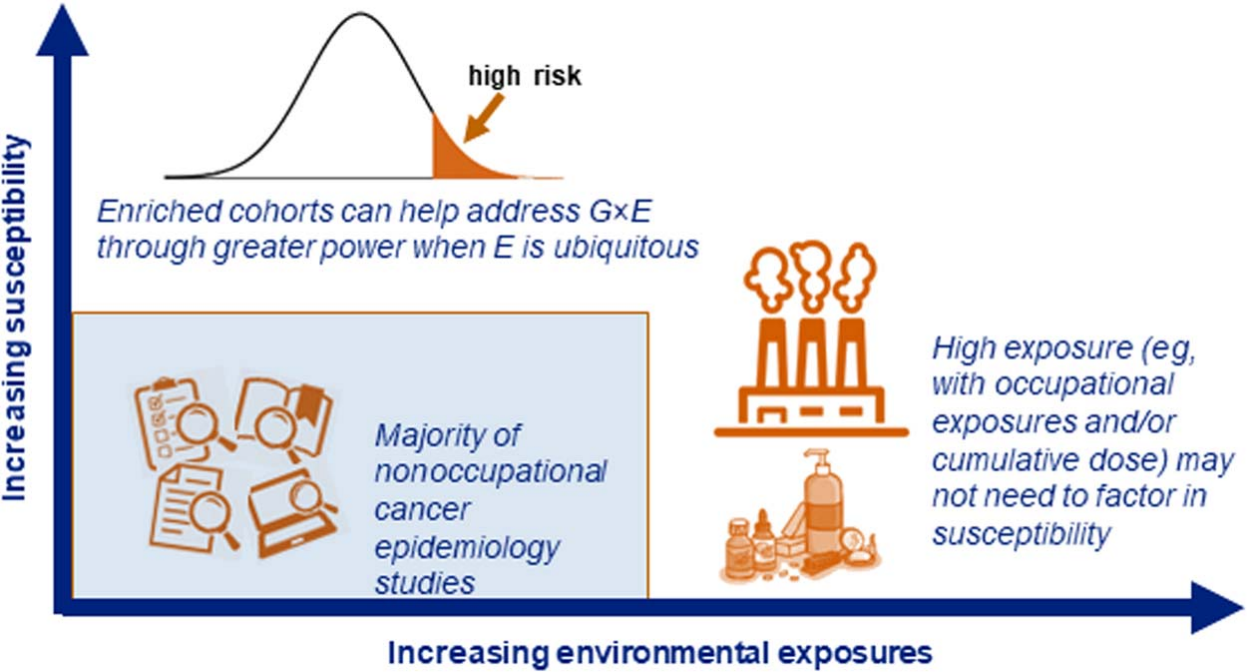
Schematic overview of cohorts enriched for cancer susceptibility, environmental exposures, or neither.

When exposures are high, such as in occupational cohorts, the signal may be strong enough that cancer susceptibility does not have to be accounted for (Figure 2, bottom right). However, when exposures are chronic but at a lower level, as in nonoccupational settings, having strong consistent signals will require accurately measuring both the exposure and cancer susceptibility (Figure 2, top right). Enriched cohorts (ie, cohort studies that oversample for individuals with underlying genetic susceptibility for cancer) are thus a useful design for studying environmental exposures.^100^ Cancer gene discovery uses the approach of enrichment to identify genes that are relevant to all but would be difficult to identify in averagerisk cohorts.^100^ In a similar way, we have used enriched cohorts to robustly examine whether modifiable factors have a different relative risk based on underlying susceptibility.^100^^‑^^105^ While enriched cohorts are oversampled for higher-risk individuals, they also include individuals at low and average risk for cancer.^100^ Therefore, enriched cohorts can be used to study environmental carcinogens across the continuum of absolute cancer risk.

### Making the case for case-control studies and other study designs

Given the aforementioned limitations of nonenriched cancer cohort studies, other study designs are needed that can further build the evidence base around the environment and cancer. This includes enriched cohorts and other novel cohort study designs (see Table 2). Cross-sectional studies and ecological studies may also be useful designs for understanding the increase in early-onset cancers over time. For example, we have used population-based registry data to describe US and global trends over time in early-onset breast and colorectal cancer incidence rates, which have informed hypotheses related to the role of reproductive patterns and obesity in these cancers.^106^^,^^107^ We have also developed methods for examining birth cohort trends in early-onset cancers (eg, see Yang et al^108^ and Yang and Terry^109^).

**Table 2:** Alternative cohort study designs for evaluating environmental carcinogens.

**Design**	**Considerations**	**Examples (not an exhaustive list)**
Cohorts enriched for cancer susceptibility Cohorts enriched for individuals with a family history of cancer or genetic susceptibility Cohorts enriched for individuals with intermediate phenotypes of cancer risk (eg, fatty liver disease, colorectal polyps, benign breast disease)	Findings may be less generalizable to average-risk populations. However, cancer family cohorts can provide data on individuals across the continuum of absolute cancer risk (see Terry et al^100^).	*Family risk cohorts:* Breast Cancer Family Registry (https://www.bcfamilyregistry.org) LEGACY Girls Study (https://www.bcfamilyregistry.org/ongoing-grants/legacy-girls-study) Sister Study (https://sisterstudy.niehs.nih.gov) Colon Cancer Family Registry (https://coloncfr.org) *Intermediate phenotypes cohorts:* Southern Liver Health Study (https://ceecr.org/cohorts/strive)
Cohorts enriched for environmental exposure Occupational cohorts Disaster-exposed cohorts Cohorts of individuals living in geographical areas with high exposure	Occupational cohort studies may suffer from bias such as the healthy worker effect.^69^^‑^^71^ Findings may be less generalizable to the general population with lower doses of environmental exposures.^72^	*Occupational cohorts:* Fire Fighter Cancer Cohort Study (https://www.ffccs.org) GuLFSTUDY (https://gulfstudy.nih.gov/en/index.html) *Disaster-exposed cohorts:* World Trade Center Health Registry (http://www.wtcregistry.org) *Geographically exposed cohorts:* Michigan Cancer and Research on the Environment Study (https://www.micares.health)
Intergenerational cohorts capturing environmental exposures during vulnerable windows of susceptibility Pregnancy cohorts Family cohorts	It may be difficult to measure cancer risk directly due to the long latency of tumorigenesis. May need to utilize intermediate markers of cancer risk (eg, DNA damage, inflammation, oxidative stress).	*Pregnancy cohorts:* DREAM Study (https://ceecr.org/cohorts/dream) Child Health and Development Studies (https://www.chdstudies.org) New York City Mothers and Newborns Study (https://tools.niehs.nih.gov/cohorts/index.cfm/main/detail/search/true/ids/c25) *Family cohorts:* 10,000 Families Study (https://ceecr.org/cohorts/10kfs)
Pooled cohort studies	Data harmonization may be challenging, including availability and harmonization of biomarker data. Existing studies available for pooling may not be racially and ethnically diverse.	*Pooled cohorts:* Premenopausal Breast Cancer Collaboration (https://pubmed.ncbi.nlm.nih.gov/28600297)

Abbreviations: DREAM, Discovering Cancer Risk from Environmental Contaminants and Maternal Child Health; GuLFSTUDY, Gulf Long-term Follow-up Study; LEGACY, Lessons in Epidemiology and Genetics of Adult Cancer from Youth.

**Figure 3:**
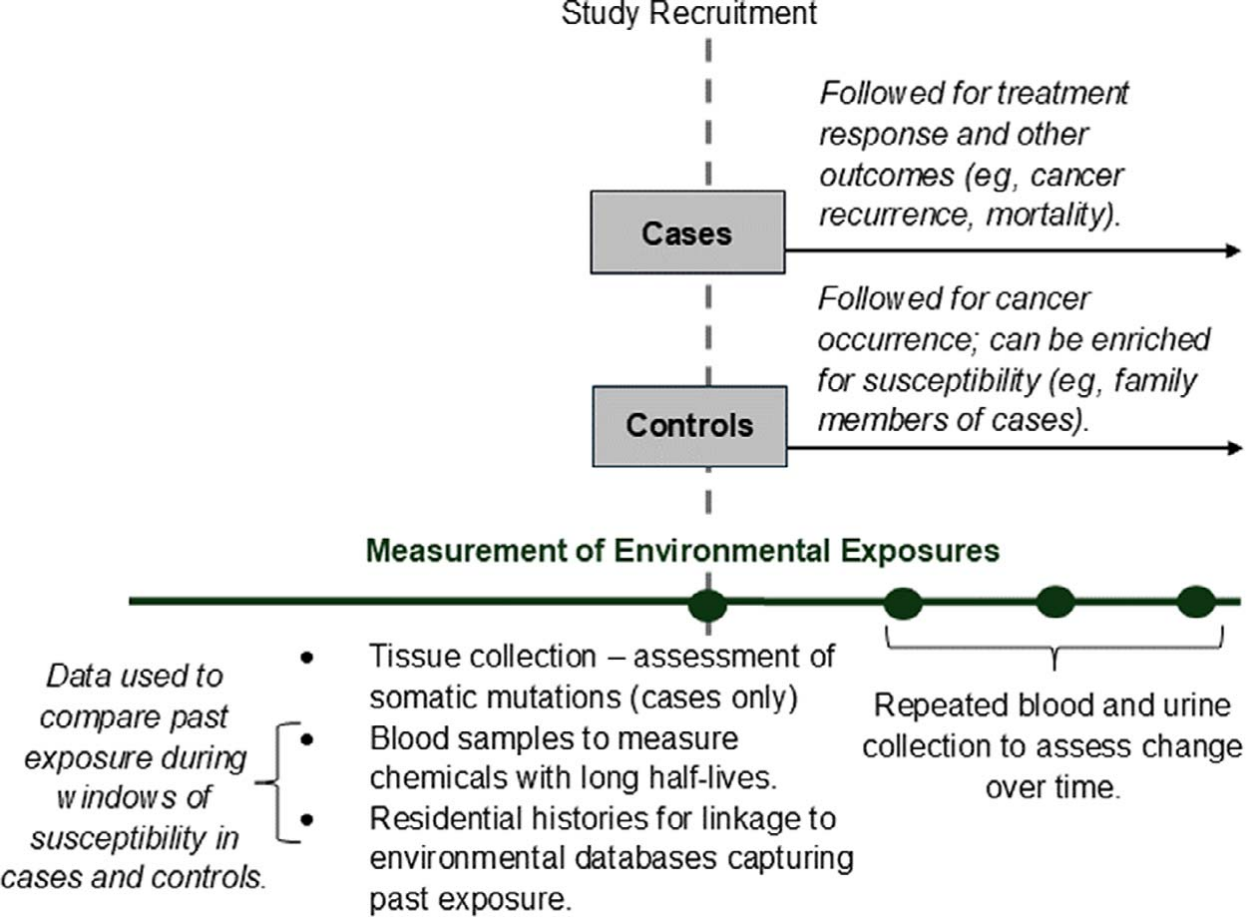
Applications of the case-control study design for studying environmental exposures and cancer.

In addition, as outlined in Figure 3, there is a role for case-control studies in environmental epidemiology studies, especially for studying early-onset cancers that have increased in incidence over time but still have low absolute rates.^16^ As described above in the “Measurement of environmental exposure constructs” section, the availability of environmental databases that can be linked to residential histories allows for the evaluation of environmental exposures prior to disease onset in cases. Some environmental chemicals also have very long half-lives and can be measured through a case-control design. Further, cases can be followed prospectively over time with repeated biospecimen collection to examine whether environmental exposures are associated with treatment response and other postdiagnosis outcomes (eg, cancer recurrence, mortality). Controls could also be prospectively followed over time for cancer occurrence, especially if controls are enriched for susceptibility (eg, family members of cancer cases). Tissue collection may also be more feasible in case-control studies than in cohort studies, allowing for the measurement of somatic mutations and their interaction with or mediation of environmental exposures in cases. Given these advantages, case-control studies should not be overlooked as a key tool for building the body of human evidence on environmental carcinogens.

## Example with BC

In this section, we use the example of BC to demonstrate why the inclusion of underlying cancer susceptibility and windows of susceptibility (WOS) in study eligibility and measurement protocols are essential for developing epidemiologic evidence on environmental exposures. BC incidence is increasing globally, particularly among women under age 50 years, and changes in reproductive patterns only partially explain these trends.^106^ The International Agency for Research on Cancer has estimated that two-thirds of cancers diagnosed before age 50 years are in women, with BC ranking first.^110^ Therefore, it is critical for global health to understand these trends.

Of the 4 most common cancers, only lung cancer has been definitively linked to environmental carcinogens, and of the other 3 (breast, prostate, and colorectal cancers), there are substantially more data available for BC. Over the past 25 years, hundreds of studies have examined associations between environmental chemicals and BC (eg, see Gammon et al^111^^,^^112^). While there is a growing body of literature to support the hypothesis that certain occupations like firefighting are associated with BC,^113^^,^^114^ most of the epidemiologic evidence remains largely inconsistent about whether any specific environmental exposure causes BC. This is in contrast to a large body of literature from laboratory studies supporting the hypothesis that many environmental exposures operate as endocrine disrupters.^115^^,^^116^ Most of the epidemiologic evidence comes from studies in average-risk populations that have not been enriched for cancer susceptibility. As we described above, this makes it challenging to identify signals from ubiquitous environmental exposures at low levels. In a recent review of 56 studies that classified cancer susceptibility through either design or analyses, we found that most studies supported a positive association between environmental exposures and BC risk.^41^ Specifically, we found that 80% (8/10) of publications from studies that enriched for women with a BC family history or early-onset BC reported a statistically significant positive association between environmental exposures and BC risk.^41^ Additionally, 74% (20/27) of publications from studies that considered gene–environment interactions reported a statistically significant positive association between environmental exposures and BC risk in women with higher cancer susceptibility.^41^ These findings support the hypothesis that environmental signals may be easier to detect in cohorts that are enriched for cancer susceptibility than in population-based cohorts. We again note that findings from enriched cohorts have relevance for individuals across the absolute cancer risk continuum, just like how genes identified in enriched studies have relevance for all, including those at average risk.^41^

Epidemiologic and mechanistic studies support the hypothesis that BC, in addition to having a long induction time like many cancers, has specific WOS related to when the breast tissue is changing in form and function.^117^^,^^118^ These WOS include the prenatal window, puberty, pregnancy and lactation, and the menopausal transition.^115^^,^^118^ Brody et al^119^ conducted a systematic review of the epidemiologic evidence on common environmental exposures and BC risk and found that only 11% of studies in the very large literature were conducted within a specific WOS. When we look at the evidence collected within these WOS, just like when we consider underlying cancer susceptibility, the evidence is much more consistently positive regarding the relationship between common environmental exposures and BC risk.^120^ These systematic reviews reveal that (1) the bulk of epidemiologic evidence on environmental carcinogens has been conducted without factoring in susceptibility based on either germline susceptibility or WOS and (2) the evidence is much more consistently positive when susceptibility is accounted for in the study design (eg, enriched cohorts, measurements collected during WOS) and/or analysis (eg, testing for gene–environment interactions).

Much stronger associations between environmental exposures and cancer risk may be observed when study designs can integrate susceptibility into the eligibility and measurement protocols. This translates into a greater ability to rule out selection, information, or confounding bias as the primary explanation for the findings.^121^ For example, when we examined the relationship between PAH exposure and BC risk in a large population-based case-control study, we found only modest support for an association overall and no clear dose–response relationship was observed.^122^ However, we found much stronger evidence of an association in subgroups of women with pathogenic variants in DNA repair genes.^123^^‑^^125^ Additionally, in a study using the Breast Cancer Family Registry (BCFR), an enriched family-based cohort, we found that the association between PAH exposure and BC risk was much stronger in individuals with greater underlying susceptibility based on BC family history (eg, an odds ratio greater than 4 for women in the highest category of absolute BC risk).^126^ We have also used the BCFR to study risk factors and their interaction with cancer susceptibility in relation to secondary cancers, mortality, and other outcomes in the survivorship cohort.^127^^‑^^132^ Further, we have leveraged the family-based design of the BCFR to conduct efficient case-control sampling within families to minimize confounding from early-life exposures, including environmental factors that are shared within families.^133^^‑^^135^ These studies illustrate the value of using enriched cohorts and leveraging data on germline susceptibility to build epidemiologic evidence of environmental carcinogens.

## Moving forward to confront the cancer burden

**Figure 4:**
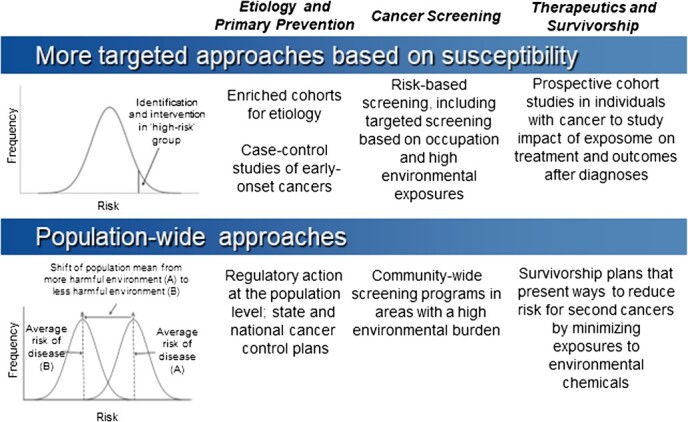
Approaches for environmental epidemiology across the cancer control continuum.

In the absence of a highly effective vaccine, like the human papillomavirus vaccine for cancers driven by human papillomavirus (eg, cervical, anal, and head and neck cancers), multiple approaches will always be needed to effectively reduce risk across the cancer control continuum. This includes reducing risk for the most common cancers, like breast, prostate, lung, and colorectal cancer. Figure 4 illustrates ways that both targeted approaches using enriched study designs and population-wide approaches can be useful for etiology and prevention, early detection and screening, and therapeutics and survivorship. In terms of etiology, we argue that reliance on existing cohorts and samples within these cohorts will not be sufficient for building the evidence base around the environment and cancer. Instead, we need to invest in new studies enriched for younger and more susceptible individuals. We must also consider returning to the case-control study design, which may be used to efficiently study the role of the environment in early-onset cancers. For example, a case-control study using residential histories and database linkages to measure exposures during the pregnancy and pubertal windows may shed light on the increase in early-onset BC over time. In addition, it will be important for new cohort and case-control studies to be carried out in diverse communities that are underrepresented in existing cancer cohorts.

While enriched studies have greater statistical power for identifying etiological evidence, prevention must be both targeted and population-wide (eg, through regulatory action at the local, state, and federal levels).^6^^,^^136^^,^^137^ Targeted and population-wide approaches are also both important for cancer screening. For example, risk-based screening strategies are being evaluated, even for cancers that have effective population-level screening programs (eg, see Esserman^138^). For cancers that do not have population-level screening, targeted screening programs for occupations or communities with higher cancer risk are essential. These initiatives will be essential for reducing cancer mortality from lung and other cancers, which is one of the important ways we can meet the Cancer Moonshot goals.^139^ Finally, we must also consider the role of the environment in treatment response and outcomes after diagnosis (eg, cancer recurrence, mortality) in populations already affected by cancer. For example, recent data support the hypothesis that air pollution may operate as a tumor promoter for lung cancer^140^ and may affect lung cancer treatment and survival through different pathways. The full impact of environmental exposures on cancer treatment and outcomes is probably underestimated given that environmental agents can cause many chronic conditions that can affect cancer outcomes, and some chronic conditions are exclusion criteria for participation in clinical trials. Therefore, the road map we have provided here for using novel and complementary measurements (eg, multi-omics platforms), along with the full hierarchy of study designs, in environmental epidemiology studies should be applied across the cancer control continuum. Only then will we be able to accurately estimate the full burden of environmental carcinogens on human health.

## Supplementary Material

Web_Material_kwae175

## Data Availability

No original data were used in this article.

## References

[1] Peters A, Nawrot TS, Baccarelli AA. Hallmarks of environmental insults. *Cell*. 2021;184(6):1455-1468. 10.1016/j.cell.2021.01.043 33657411 PMC9396710

[2] Yilmaz B, Terekeci H, Sandal S, et al. Endocrine disrupting chemicals: exposure, effects on human health, mechanism of action, models for testing and strategies for prevention. *Rev Endocr Metab Disord*. 2020;21(1):127-147. 10.1007/s11154-019-09521-z 31792807

[3] Turner MC, Andersen ZJ, Baccarelli A, et al. Outdoor air pollution and cancer: an overview of the current evidence and public health recommendations. *CA Cancer J Clin*. 2020;70(6):460-479. 10.3322/caac.21632 PMC7904962 32964460

[4] International Agency for Research on Cancer . Agents classified by the IARC Monographs, volumes 1-136. Accessed July 17, 2023. https://monographs.iarc.who.int/agents-classified-by-the-iarc/

[5] International Agency for Research on Cancer . Preamble to the *IARC Monographs* (amended January 2019). 2019. Accessed July 17, 2023. https://monographs.iarc.who.int/cards_page/preamble-monographs/

[6] Division of Cancer Prevention and Control, Centers for Disease Control and Prevention . About comprehensive cancer control programs. 2023. Updated August 9, 2023. Accessed November 15, 2023. https://www.cdc.gov/comprehensive-cancer-control/about/programs.html?CDC_AAref_Val=https://www.cdc.gov/cancer/ncccp/ccc_plans.htm

[7] Doll R, Peto R. The causes of cancer: quantitative estimates of avoidable risks of cancer in the United States today. *J Natl Cancer Inst*. 1981;66(6):1191-1308. 10.1093/jnci/66.6.1192 7017215

[8] GBD 2019 Cancer Risk Factors Collaborators . The global burden of cancer attributable to risk factors, 2010–19: a systematic analysis for the Global Burden of Disease Study 2019. *Lancet*. 2022;400(10352):563-591. 10.1016/S0140-6736(22)01438-6 35988567 PMC9395583

[9] Blot WJ, Tarone RE. Doll and Peto’s quantitative estimates of cancer risks: holding generally true for 35 years. *J Natl Cancer Inst*. 2015;107(4):djv044. 10.1093/jnci/djv044 25739419

[10] Song M, Giovannucci EL. RE: Doll and Peto’s quantitative estimates of cancer risks: holding generally true for 35 years. *J Natl Cancer Inst*. 2015;7(10):djv240. 10.1093/jnci/djv240 26271254

[11] Spirtas R, Heineman EF, Bernstein L, et al. Malignant mesothelioma: attributable risk of asbestos exposure. *Occup Environ Med*. 1994;51(12):804-811. 10.1136/oem.51.12.804 7849863 PMC1128120

[12] Labrèche F, Kim J, Song C, et al. The current burden of cancer attributable to occupational exposures in Canada. *Prev Med*. 2019;122:128-139. 10.1016/j.ypmed.2019.03.016 31078166

[13] Patrick DL, Cheadle A, Thompson DC, et al. The validity of self-reported smoking: a review and meta-analysis. *Am J Public Health*. 1994;84(7):1086-1093. 10.2105/AJPH.84.7.1086 8017530 PMC1614767

[14] Wong SL, Shields M, Leatherdale S, et al. Assessment of validity of self-reported smoking status. *Health Rep*. 2012;23(1):47-53. 22590805

[15] Wild CP . Environmental exposure measurement in cancer epidemiology. *Mutagenesis*. 2009;24(2):117-125. 10.1093/mutage/gen061 19033256 PMC2720689

[16] Kehm RD, Yang W, Tehranifar P, et al. 40 years of change in age- and stage-specific cancer incidence rates in US women and men. *JNCI Cancer Spectr*. 2019;3(3):pkz038. 10.1093/jncics/pkz038 31414075 PMC6686848

[17] Office of the Surgeon General, US Public Health Service . Smoking Cessation: A Report of the Surgeon General. US Department of Health and Human Services; 2020. 32255575

[18] Dai X, Gakidou E, Lopez AD. Evolution of the global smoking epidemic over the past half century: strengthening the evidence base for policy action. *Tob Control*. 2022;31(2):129-137. 10.1136/tobaccocontrol-2021-056535 35241576

[19] Siegel RL, Miller KD, Wagle NS, et al. Cancer statistics, 2023. *CA Cancer J Clin*. 2023;73(1):17-48. 10.3322/caac.21763 36633525

[20] Ruiz D, Becerra M, Jagai JS, et al. Disparities in environmental exposures to endocrine-disrupting chemicals and diabetes risk in vulnerable populations. *Diabetes Care*. 2018;41(1):193-205. 10.2337/dc16-2765 29142003 PMC5741159

[21] Richmond-Bryant J, Mikati I, Benson AF, et al. Disparities in distribution of particulate matter emissions from US coal-fired power plants by race and poverty status after accounting for reductions in operations between 2015 and 2017. *Am J Public Health*. 2020;110(5):655-661. 10.2105/AJPH.2019.305558 32191524 PMC7144440

[22] Fong KC, Bell ML. Do fine particulate air pollution (PM_2.5_) exposure and its attributable premature mortality differ for immigrants compared to those born in the United States? *Environ Res*. 2020;196:110387. 10.1016/j.envres.2020.110387 33129853 PMC8079555

[23] Duty SM, Ackerman RM, Calafat AM, et al. Personal care product use predicts urinary concentrations of some phthalate monoesters. *Environ Health Perspect*. 2005;113(11):1530-1535. 10.1289/ehp.8083 16263507 PMC1310914

[24] James-Todd T, Terry MB, Rich-Edwards J, et al. Childhood hair product use and earlier age at menarche in a racially diverse study population: a pilot study. *Ann Epidemiol*. 2011;21(6):461-465. 10.1016/j.annepidem.2011.01.009 21421329 PMC4116338

[25] James-Todd T, Senie R, Terry MB. Racial/ethnic differences in hormonally-active hair product use: a plausible risk factor for health disparities. *J Immigr Minor Health*. 2012;14(3):506-511. 10.1007/s10903-011-9482-5 21626298

[26] Branch F, Woodruff TJ, Mitro SD, et al. Vaginal douching and racial/ethnic disparities in phthalates exposures among reproductive-aged women: National Health and Nutrition Examination Survey 2001–2004. *Environ Health*. 2015;14(1):57. 10.1186/s12940-015-0043-6 26174070 PMC4502470

[27] Hicken MT, Gee GC, Morenoff J, et al. A novel look at racial health disparities: the interaction between social disadvantage and environmental health. *Am J Public Health*. 2012;102(12):2344-2351. 10.2105/AJPH.2012.300774 23078461 PMC3519308

[28] Alcala E, Brown P, Capitman JA, et al. Cumulative impact of environmental pollution and population vulnerability on pediatric asthma hospitalizations: a multilevel analysis of CalEnviroScreen. *Int J Environ Res Public Health*. 2019;16(15):2683. 10.3390/ijerph16152683 31357578 PMC6696276

[29] Morenoff JD, House JS, Hansen BB, et al. Understanding social disparities in hypertension prevalence, awareness, treatment, and control: the role of neighborhood context. *Soc Sci Med*. 2007;65(9):1853-1866. 10.1016/j.socscimed.2007.05.038 17640788 PMC2705439

[30] Sung H, Ferlay J, Siegel RL, et al. Global cancer statistics 2020: GLOBOCAN estimates of incidence and mortality worldwide for 36 cancers in 185 countries. *CA Cancer J Clin*. 2021;71(3):209-249. 10.3322/caac.21660 33538338

[31] Brown JR, Thornton JL. Percivall Pott (1714-1788) and chimney sweepers’ cancer of the scrotum. *Br J Ind Med*. 1957;14(1):68-70. 10.1136/oem.14.1.68 13396156 PMC1037746

[32] Polednak AP . Bone cancer among female radium dial workers. Latency periods and incidence rates by time after exposure: *brief communication*. *J Natl Cancer Inst*. 1978;60(1):77-82. 10.1093/jnci/60.1.77 628024

[33] Fry SA . Studies of U.S. radium dial workers: an epidemiological classic. *Radiat Res*. 1998;150(5 suppl):S21-S29. 10.2307/3579805 9806606

[34] Siemiatycki J, Dewar R, Richardson L. Costs and statistical power associated with five methods of collecting occupation exposure information for population-based case-control studies. *Am J Epidemiol*. 1989;130(6):1236-1246. 10.1093/oxfordjournals.aje.a115452 2589314

[35] Nieuwenhuijsen MJ . Design of exposure questionnaires for epidemiological studies. *Occup Environ Med*. 2005;62(4):272-280. 10.1136/oem.2004.015206 15778263 PMC1740990

[36] Perera FP, Weinstein IB. Molecular epidemiology and carcinogen-DNA adduct detection: new approaches to studies of human cancer causation. *J Chronic Dis*. 1982;35(7):581-600. 10.1016/0021-9681(82)90078-9 6282919

[37] Perera FP, Weinstein IB. Molecular epidemiology: recent advances and future directions. *Carcinogenesis*. 2000;21(3):517-524. 10.1093/carcin/21.3.517 10688872

[38] Best LG, Garcia-Esquinas E, Yeh JL, et al. Association of diabetes and cancer mortality in American Indians: the Strong Heart Study. *Cancer Causes Control*. 2015;26(11):1551-1560. 10.1007/s10552-015-0648-7 26250516 PMC4596901

[39] Dennis KK, Auerbach SS, Balshaw DM, et al. The importance of the biological impact of exposure to the concept of the exposome. *Environ Health Perspect*. 2016;124(10):1504-1510. 10.1289/EHP140 27258438 PMC5047763

[40] Jung KH, Perzanowski M, Rundle A, et al. Polycyclic aromatic hydrocarbon exposure, obesity and childhood asthma in an urban cohort. *Environ Res*. 2014;128:35-41. 10.1016/j.envres.2013.12.002 24407477 PMC3912566

[41] Zeinomar N, Oskar S, Kehm RD, et al. Environmental exposures and breast cancer risk in the context of underlying susceptibility: a systematic review of the epidemiological literature. *Environ Res*. 2020;187:109346. 10.1016/j.envres.2020.109346 32445942 PMC7314105

[42] Parada H Jr, Gammon MD, Ettore HL, et al. Urinary concentrations of environmental phenols and their associations with breast cancer incidence and mortality following breast cancer. *Environ Int*. 2019;130:104890. 10.1016/j.envint.2019.05.084 31228785 PMC6679996

[43] Wu HC, Brennan LA, Goldberg M, et al. Influence of pubertal development on urinary oxidative stress biomarkers in adolescent girls in the New York LEGACY cohort. *Free Radic Res*. 2020;54(6):431-441. 10.1080/10715762.2020.1798001 32686531 PMC7731215

[44] Karagas MR, Tosteson TD, Blum J, et al. Measurement of low levels of arsenic exposure: a comparison of water and toenail concentrations. *Am J Epidemiol*. 2000;152(1):84-90. 10.1093/aje/152.1.84 10901333

[45] Strickland P, Kang D, Sithisarankul P. Polycyclic aromatic hydrocarbon metabolites in urine as biomarkers of exposure and effect. *Environ Health Perspect*. 1996;104(suppl 5):927-932. 10.1289/ehp.96104s5927 8933036 PMC1469694

[46] Jacob J, Seidel A. Biomonitoring of polycyclic aromatic hydrocarbons in human urine. *J Chromatogr B Analyt Technol Biomed Life Sci*. 2002;778(1-2):31-47. 10.1016/S0378-4347(01)00467-4 12376115

[47] Gammon MD, Wolff MS, Neugut AI, et al. Treatment for breast cancer and blood levels of chlorinated hydrocarbons. *Cancer Epidemiol Biomarkers Prev*. 1996;5(6):467-471. 8781744

[48] La Merrill M, Emond C, Kim MJ, et al. Toxicological function of adipose tissue: focus on persistent organic pollutants. *Environ Health Perspect*. 2013;121(2):162-169. 10.1289/ehp.1205485 23221922 PMC3569688

[49] Schroeder JC, Conway K, Li Y, et al. *p53* mutations in bladder cancer: evidence for exogenous *versus* endogenous risk factors. *Cancer Res*. 2003;63(21):7530-7538. 14612556

[50] Landi MT, Synnott NC, Rosenbaum J, et al. Tracing lung cancer risk factors through mutational signatures in never-smokers. *Am J Epidemiol*. 2021;190(6):962-976. 10.1093/aje/kwaa234 33712835 PMC8316614

[51] Gharibvand L, Shavlik D, Ghamsary M, et al. The association between ambient fine particulate air pollution and lung cancer incidence: results from the AHSMOG-2 study. *Environ Health Perspect*. 2017;125(3):378-384. 10.1289/EHP124 27519054 PMC5332173

[52] Echeverri M, Anderson D, Nápoles AM, et al. Cancer health literacy and willingness to participate in cancer research and donate bio-specimens. *Int J Environ Res Public Health*. 2018;15(10):2091. 10.3390/ijerph15102091 30249985 PMC6211072

[53] Bartell SM, Griffith WC, Faustman EM. Temporal error in biomarker-based mean exposure estimates for individuals. *J Expo Anal Environ Epidemiol*. 2004;14(2):173-179. 10.1038/sj.jea.7500311 15014548

[54] Escher BI, Stapleton HM, Schymanski EL. Tracking complex mixtures of chemicals in our changing environment. *Science*. 2020;367(6476):388-392. 10.1126/science.aay6636 31974244 PMC7153918

[55] O’Connell SG, Kincl LD, Anderson KA. Silicone wristbands as personal passive samplers. *Environ Sci Technol*. 2014;48(6):3327-3335. 10.1021/es405022f 24548134 PMC3962070

[56] Levasseur JL, Hoffman K, Herkert NJ, et al. Characterizing firefighter’s exposure to over 130 SVOCs using silicone wristbands: a pilot study comparing on-duty and off-duty exposures. *Sci Total Environ*. 2022;834:155237. 10.1016/j.scitotenv.2022.155237 35447169 PMC9728008

[57] Vermeulen R, Schymanski EL, Barabási AL, et al. The exposome and health: where chemistry meets biology. *Science*. 2020;367(6476):392-396. 10.1126/science.aay3164 31974245 PMC7227413

[58] Centers for Disease Control and Prevention . National Environmental Public Health Tracking Network. 2023. Reviewed May 31, 2023. Accessed July 17, 2023. https://ephtracking.cdc.gov/

[59] Environmental Protection Agency . EJScreen: Environmental Justice Screening and Mapping Tool. 2023. Updated September 9, 2024. Accessed July 17, 2023. https://www.epa.gov/ejscreen

[60] New York State Department of Health . Environmental Public Health Tracking. 2023. Revised September 2024. Accessed July 17, 2023. https://www.health.ny.gov/environmental/public_health_tracking/

[61] van Donkelaar A, Martin RV, Spurr RJ, et al. High-resolution satellite-derived PM_2.5_ from optimal estimation and geographically weighted regression over North America. *Environ Sci Technol*. 2015;49(17):10482-10491. 10.1021/acs.est.5b02076 26261937

[62] Di Q, Kloog I, Koutrakis P, et al. Assessing PM_2.5_ exposures with high spatiotemporal resolution across the continental United States. *Environ Sci Technol*. 2016;50(9):4712-4721. 10.1021/acs.est.5b06121 27023334 PMC5761665

[63] Kloog I, Chudnovsky AA, Just AC, et al. A new hybrid spatio-temporal model for estimating daily multi-year PM_2.5_ concentrations across northeastern USA using high resolution aerosol optical depth data. *Atmos Environ*. 1994;95(95):581-590. 10.1016/j.atmosenv.2014.07.014 PMC5621749 28966552

[64] Di Q, Amini H, Shi L, et al. An ensemble-based model of PM_2.5_ concentration across the contiguous United States with high spatiotemporal resolution. *Environ Int*. 2019;130:104909. 10.1016/j.envint.2019.104909 31272018 PMC7063579

[65] Kim SY, Bechle M, Hankey S, et al. Concentrations of criteria pollutants in the contiguous U.S., 1979–2015: role of prediction model parsimony in integrated empirical geographic regression. *PloS One*. 2020;15(2):e0228535. 10.1371/journal.pone.0228535 32069301 PMC7028280

[66] Shaddick G, Thomas ML, Green A, et al. Data integration model for air quality: a hierarchical approach to the global estimation of exposures to ambient air pollution. *J R Stat Soc Ser C Appl Stat*. 2018;67(1):231-253. 10.1111/rssc.12227

[67] Hall ES, Eyth AM, Phillips SB, et al. Hierarchical Bayesian Model (HBM)-Derived Estimates of Air Quality for 2008: Annual Report. Environmental Protection Agency; 2020.

[68] Shkembi A, Smith LM, Neitzel RL. Linking environmental injustices in Detroit, MI to institutional racial segregation through historical federal redlining. *J Expo Sci Environ Epidemiol*. 2022;34(3):389-398. 10.1038/s41370-022-00512-y 36544051 PMC11222141

[69] Li CY, Sung FC. A review of the healthy worker effect in occupational epidemiology. *Occup Med*. 1999;49(4):225-229. 10.1093/occmed/49.4.225 10474913

[70] Chowdhury R, Shah D, Payal AR. Healthy worker effect phenomenon: revisited with emphasis on statistical methods—a review. *Indian J Occup Environ Med*. 2017;21(1):2-8. 10.4103/ijoem.IJOEM_53_16 29391741 PMC5763838

[71] Shah D . Healthy worker effect phenomenon. *Indian J Occup Environ Med*. 2009;13(2):77-79. 10.4103/0019-5278.55123 20386623 PMC2847330

[72] Iwasaki M, Yamamoto S, Otani T, et al. Generalizability of relative risk estimates from a well-defined population to a general population. *Eur J Epidemiol*. 2006;21(4):253-262. 10.1007/s10654-006-0004-z 16685575

[73] Hohenadel K, Raj P, Demers PA, et al. The inclusion of women in studies of occupational cancer: a review of the epidemiologic literature from 1991–2009. *Am J Ind Med*. 2015;58(3):276-281. 10.1002/ajim.22424 25678456

[74] Hoffmann S, Laurier D, Rage E, et al. Shared and unshared exposure measurement error in occupational cohort studies and their effects on statistical inference in proportional hazards models. *PloS One*. 2018;13(2):e0190792. 10.1371/journal.pone.0190792 29408862 PMC5800563

[75] Pearce N, Checkoway H, Kriebel D. Bias in occupational epidemiology studies. *Occup Environ Med*. 2007;64(8):562-568. 10.1136/oem.2006.026690 17053019 PMC2078501

[76] Blair A, Stewart P, Lubin JH, et al. Methodological issues regarding confounding and exposure misclassification in epidemiological studies of occupational exposures. *Am J Ind Med*. 2007;50(3):199-207. 10.1002/ajim.20281 17096363

[77] Rim K-T . Exposure of chemical mixtures at work and their application to the prevention of occupational disease. *Toxicol Environ Health Sci*. 2021;13(2):91-99. 10.1007/s13530-021-00087-5

[78] Fujishiro K, Xu J, Gong F. What does “occupation” represent as an indicator of socioeconomic status?: exploring occupational prestige and health. *Soc Sci Med*. 2010;71(12):2100-2107. 10.1016/j.socscimed.2010.09.026 21041009

[79] Borghi F, Mazzucchelli LA, Campagnolo D, et al. Retrospective exposure assessment methods used in occupational human health risk assessment: a systematic review. *Int J Environ Res Public Health*. 2020;17(17):6190. 10.3390/ijerph17176190 32858967 PMC7504303

[80] Koskela RS, Kolari PJ, Järvinen E, et al. Completeness of occupational history and occurrences of work-related diseases. *Scand J Work Environ Health*. 1984;10(6):455-459. 10.5271/sjweh.2298 6535248

[81] Hernán MA, Hernández-Díaz S, Robins JM. A structural approach to selection bias. *Epidemiology*. 2004;15(5):615-625. 10.1097/01.ede.0000135174.63482.43 15308962

[82] Schulz KF, Grimes DA. Case-control studies: research in reverse. *Lancet*. 2002;359(9304):431-434. 10.1016/S0140-6736(02)07605-5 11844534

[83] Fewell Z, Davey Smith G, Sterne JA. The impact of residual and unmeasured confounding in epidemiologic studies: a simulation study. *Am J Epidemiol*. 2007;166(6):646-655. 10.1093/aje/kwm165 17615092

[84] Boffetta P . Sources of bias, effect of confounding in the application of biomarkers to epidemiological studies. *Toxicol Lett*. 1995;77(1-3):235-238. 10.1016/0378-4274(95)03301-7 7618144

[85] Ensor JE . Biomarker validation: common data analysis concerns. *Oncologist*. 2014;19(8):886-891. 10.1634/theoncologist.2014-0061 25001264 PMC4122484

[86] Tworoger SS, Hankinson SE. Use of biomarkers in epidemiologic studies: minimizing the influence of measurement error in the study design and analysis. *Cancer Causes Control*. 2006;17(7):889-899. 10.1007/s10552-006-0035-5 16841256

[87] Patel CJ, Kerr J, Thomas DC, et al. Opportunities and challenges for environmental exposure assessment in population-based studies. *Cancer Epidemiol Biomarkers Prev*. 2017;26(9):1370-1380. 10.1158/1055-9965.EPI-17-0459 28710076 PMC5581729

[88] de Vocht F, Cherry N, Wakefield J. A Bayesian mixture modeling approach for assessing the effects of correlated exposures in case-control studies. *J Expo Sci Environ Epidemiol*. 2012;22(4):352-360. 10.1038/jes.2012.22 22588215

[89] Calafat AM, Longnecker MP, Koch HM, et al. Optimal exposure biomarkers for nonpersistent chemicals in environmental epidemiology. *Environ Health Perspect*. 2015;123(7):A166-A168. 10.1289/ehp.1510041 26132373 PMC4492274

[90] Hwa Yun B, Guo J, Bellamri M, et al. DNA adducts: formation, biological effects, and new biospecimens for mass spectrometric measurements in humans. *Mass Spectrom Rev*. 2020;39(1-2):55-82. 10.1002/mas.21570 29889312 PMC6289887

[91] Perera F, Brenner D, Jeffrey A, et al. DNA adducts and related biomarkers in populations exposed to environmental carcinogens. *Environ Health Perspect*. 1992;98:133-137. 10.1289/ehp.9298133 1486841 PMC1519595

[92] Elliott P, Wartenberg D. Spatial epidemiology: current approaches and future challenges. *Environ Health Perspect*. 2004;112(9):998-1006. 10.1289/ehp.6735 15198920 PMC1247193

[93] Dummer TJ . Health geography: supporting public health policy and planning. *CMAJ*. 2008;178(9):1177-1180. 10.1503/cmaj.071783 18427094 PMC2292766

[94] Hasin Y, Seldin M, Lusis A. Multi-omics approaches to disease. *Genome Biol*. 2017;18(1):83. 10.1186/s13059-017-1215-1 28476144 PMC5418815

[95] Karczewski KJ, Snyder MP. Integrative omics for health and disease. *Nat Rev Genet*. 2018;19(5):299-310. 10.1038/nrg.2018.4 29479082 PMC5990367

[96] Smith GD, Ebrahim S. Mendelian randomization: prospects, potentials, and limitations. *Int J Epidemiol*. 2004;33(1):30-42. 10.1093/ije/dyh132 15075143

[97] Division of Cancer Control and Population Sciences, National Cancer Institute . CEDCD: Cancer Epidemiology Descriptive Cohort Database. 2023. Accessed July 17, 2023. https://cedcd.nci.nih.gov/

[98] Clarke MA, Joshu CE. Early life exposures and adult cancer risk. *Epidemiol Rev*. 2017;39(1):11-27. 10.1093/epirev/mxx004 28407101 PMC5858036

[99] Qing T, Mohsen H, Marczyk M, et al. Germline variant burden in cancer genes correlates with age at diagnosis and somatic mutation burden. *Nat Commun*. 2020;11(1):2438. 10.1038/s41467-020-16293-7 32415133 PMC7228928

[100] Terry MB, Phillips KA, Daly MB, et al. Cohort profile: the breast cancer Prospective Family Study Cohort (ProF-SC). *Int J Epidemiol*. 2016;45(3):683-692. 10.1093/ije/dyv118 26174520 PMC5005937

[101] Hopper JL, Dite GS, MacInnis RJ, et al. Age-specific breast cancer risk by body mass index and familial risk: Prospective Family Study Cohort (ProF-SC). *Breast Cancer Res*. 2018;20(1):1-11. 10.1186/s13058-018-1056-1 30390716 PMC6215632

[102] Kehm RD, Genkinger JM, MacInnis RJ, et al. Recreational physical activity is associated with reduced breast cancer risk in adult women at high risk for breast cancer: a cohort study of women selected for familial and genetic risk. *Cancer Res*. 2020;80(1):116-125. 10.1158/0008-5472.CAN-19-1847 31578201 PMC7236618

[103] Kehm RD, Hopper JL, John EM, et al. Regular use of aspirin and other non-steroidal anti-inflammatory drugs and breast cancer risk for women at familial or genetic risk: a cohort study. *Breast Cancer Res*. 2019;21(1):1-13. 10.1186/s13058-019-1135-y 30999962 PMC6471793

[104] Terry MB, Daly MB, Phillips KA, et al. Risk-reducing oophorectomy and breast cancer risk across the spectrum of familial risk. *J Natl Cancer Inst*. 2019;111(3):331-334. 10.1093/jnci/djy182 30496449 PMC6410936

[105] Zeinomar N, Knight JA, Genkinger JM, et al. Alcohol consumption, cigarette smoking, and familial breast cancer risk: findings from the Prospective Family Study Cohort (ProF-SC). *Breast Cancer Res*. 2019;21(1):1-14. 10.1186/s13058-019-1213-1 31779655 PMC6883541

[106] Lima SM, Kehm RD, Terry MB. Global breast cancer incidence and mortality trends by region, age-groups, and fertility patterns. *EClinicalMedicine*. 2021;38:100985. 10.1016/j.eclinm.2021.100985 34278281 PMC8271114

[107] Lima SM, Kehm RD, Swett K, et al. Trends in parity and breast cancer incidence in US women younger than 40 years from 1935 to 2015. *JAMA Netw Open*. 2020;3(3):e200929. 10.1001/jamanetworkopen.2020.0929 32167569 PMC7070232

[108] Yang W, Kehm RD, Terry MB. Survival model methods for analyses of cancer incidence trends in young adults. *Stat Med*. 2020;39(7):1011-1024. 10.1002/sim.8458 32022306 PMC7288576

[109] Yang W, Terry MB. Do temporal trends in cancer incidence reveal organ system connections for cancer etiology? *Epidemiology*. 2020;31(4):595-598. 10.1097/EDE.0000000000001192 32221269 PMC7269825

[110] Vaccarella S, Ginsburg O, Bray F. Gender inequalities in cancer among young adults. *Lancet Oncol*. 2021;22(2):166-167. 10.1016/S1470-2045(21)00001-2 33539738

[111] Gammon MD, Santella RM, Neugut AI, et al. Environmental toxins and breast cancer on Long Island. I. Polycyclic aromatic hydrocarbon DNA adducts. *Cancer Epidemiol Biomarkers Prev*. 2002;11(8):677-685 Online ISSN: 1538-7755. 12163319

[112] Gammon MD, Neugut AI, Santella RM, et al. The Long Island Breast Cancer Study Project: description of a multi-institutional collaboration to identify environmental risk factors for breast cancer. *Breast Cancer Res Treat*. 2002;74(3):235-254. 10.1023/A:1016387020854 12206514

[113] Trowbridge J, Gerona RR, Lin T, et al. Exposure to perfluoroalkyl substances in a cohort of women firefighters and office workers in San Francisco. *Environ Sci Technol*. 2020;54(6):3363-3374. 10.1021/acs.est.9b05490 32100527 PMC7244264

[114] Clarity C, Trowbridge J, Gerona R, et al. Associations between polyfluoroalkyl substance and organophosphate flame retardant exposures and telomere length in a cohort of women firefighters and office workers in San Francisco. *Environ Health*. 2021;20(1):97. 10.1186/s12940-021-00778-z 34454526 PMC8403436

[115] Kay JE, Cardona B, Rudel RA, et al. Chemical effects on breast development, function, and cancer risk: existing knowledge and new opportunities. *Curr Environ Health Rep*. 2022;9(4):535-562. 10.1007/s40572-022-00376-2 35984634 PMC9729163

[116] Fenton SE, Birnbaum LS. Timing of environmental exposures as a critical element in breast cancer risk. *J Clin Endocrinol Metab*. 2015;100(9):3245-3250. 10.1210/jc.2015-2848 26214118 PMC4570175

[117] Biro FM, Deardorff J. Identifying opportunities for cancer prevention during preadolescence and adolescence: puberty as a window of susceptibility. *J Adolesc Health*. 2013;52(5 suppl):S15-S20. 10.1016/j.jadohealth.2012.09.019 23601607 PMC4037133

[118] Russo J . The windows of susceptibility to breast cancer. In: *The Pathobiology of Breast Cancer*. Springer International Publishing; 2016:1-20.

[119] Rodgers KM, Udesky JO, Rudel RA, et al. Environmental chemicals and breast cancer: an updated review of epidemiological literature informed by biological mechanisms. *Environ Res*. 2018;160:152-182. 10.1016/j.envres.2017.08.045 28987728

[120] Terry MB, Michels KB, Brody JG, et al. Environmental exposures during windows of susceptibility for breast cancer: a framework for prevention research. *Breast Cancer Res*. 2019;21(1):1-16. 10.1186/s13058-019-1168-2 31429809 PMC6701090

[121] Shapiro S . Bias in the evaluation of low-magnitude associations: an empirical perspective. *Am J Epidemiol*. 2000;151(10):939-945. 10.1093/oxfordjournals.aje.a010135 10853631

[122] Gammon MD, Sagiv SK, Eng SM, et al. Polycyclic aromatic hydrocarbon-DNA adducts and breast cancer: a pooled analysis. *Arch Environ Health*. 2004;59(12):640-649. 10.1080/00039890409602948 16789472 PMC4277204

[123] Terry MB, Gammon MD, Zhang FF, et al. Polymorphism in the DNA repair gene *XPD*, polycyclic aromatic hydrocarbon-DNA adducts, cigarette smoking, and breast cancer risk. *Cancer Epidemiol Biomarkers Prev*. 2004;13(12):2053-2058. 10.1158/1055-9965.2053.13.12 15598760

[124] Shen J, Gammon MD, Terry MB, et al. Polymorphisms in *XRCC1* modify the association between polycyclic aromatic hydrocarbon-DNA adducts, cigarette smoking, dietary antioxidants, and breast cancer risk. *Cancer Epidemiol Biomarkers Prev*. 2005;14(2):336-342. 10.1158/1055-9965.EPI-04-0414 15734955

[125] Crew KD, Gammon MD, Terry MB, et al. Polymorphisms in nucleotide excision repair genes, polycyclic aromatic hydrocarbon-DNA adducts, and breast cancer risk. *Cancer Epidemiol Biomarkers Prev*. 2007;16(10):2033-2041. 10.1158/1055-9965.EPI-07-0096 17932351

[126] Shen J, Liao Y, Hopper JL, et al. Dependence of cancer risk from environmental exposures on underlying genetic susceptibility: an illustration with polycyclic aromatic hydrocarbons and breast cancer. *Br J Cancer*. 2017;116(9):1229-1233. 10.1038/bjc.2017.81 28350789 PMC5418454

[127] Kehm RD, MacInnis RJ, John EM, et al. Recreational physical activity and outcomes after breast cancer in women at high familial risk. *JNCI Cancer Spectr*. 2021;5(6):pkab090. 10.1093/jncics/pkab090 34950851 PMC8692829

[128] Haslam DE, John EM, Knight JA, et al. Diet quality and all-cause mortality in women with breast cancer from the Breast Cancer Family Registry. *Cancer Epidemiol Biomarkers Prev*. 2023;32(5):678-686. 10.1158/1055-9965.EPI-22-1198 36857773 PMC10066732

[129] Zeinomar N, Thai A, Cloud AJ, et al. Alcohol consumption and breast cancer-specific and all-cause mortality in women diagnosed with breast cancer at the New York site of the Breast Cancer Family Registry. *PloS One*. 2017;12(12):e0189118. 10.1371/journal.pone.0189118 29244822 PMC5731703

[130] Zhang FF, Haslam DE, Terry MB, et al. Dietary isoflavone intake and all-cause mortality in breast cancer survivors: the Breast Cancer Family Registry. *Cancer*. 2017;123(11):2070-2079. 10.1002/cncr.30615 28263368 PMC5444962

[131] Phillips KA, Milne RL, West DW, et al. Prediagnosis reproductive factors and all-cause mortality for women with breast cancer in the Breast Cancer Family Registry. *Cancer Epidemiol Biomarkers Prev*. 2009;18(6):1792-1797. 10.1158/1055-9965.EPI-08-1014 19505912 PMC2746957

[132] Chang ET, Milne RL, Phillips KA, et al. Family history of breast cancer and all-cause mortality after breast cancer diagnosis in the Breast Cancer Family Registry. *Breast Cancer Res Treat*. 2009;117(1):167-176. 10.1007/s10549-008-0255-3 19034644 PMC2728159

[133] Terry MB, Knight JA, Zablotska L, et al. Alcohol metabolism, alcohol intake, and breast cancer risk: a sister-set analysis using the Breast Cancer Family Registry. *Breast Cancer Res Treat*. 2007;106(2):281-288. 10.1007/s10549-007-9498-7 17268812

[134] Milne RL, John EM, Knight JA, et al. The potential value of sibling controls compared with population controls for association studies of lifestyle-related risk factors: an example from the Breast Cancer Family Registry. *Int J Epidemiol*. 2011;40(5):1342-1354. 10.1093/ije/dyr110 21771852 PMC3204209

[135] Zhang FF, John EM, Knight JA, et al. Total energy intake and breast cancer risk in sisters: the Breast Cancer Family Registry. *Breast Cancer Res Treat*. 2013;137(2):541-551. 10.1007/s10549-012-2342-8 23225141 PMC4032289

[136] Environmental Protection Agency . Summary of the Clean Water Act. 2023. Updated June 12, 2024. Accessed November 15, 2023. https://www.epa.gov/laws-regulations/summary-clean-water-act

[137] Environmental Protection Agency . Summary of the Clean Air Act. 2023. Updated July 31, 2024. Accessed November 15, 2023. https://www.epa.gov/laws-regulations/summary-clean-air-act

[138] Esserman LJ, Anton-Culver H, Borowsky A, et al. The WISDOM Study: breaking the deadlock in the breast cancer screening debate. *NPJ Breast Cancer*. 2017;3(1):34. 10.1038/s41523-017-0035-5 28944288 PMC5597574

[139] Shiels MS, Lipkowitz S, Campos NG, et al. Opportunities for achieving the Cancer Moonshot goal of a 50% reduction in cancer mortality by 2047. *Cancer Discov*. 2023;13(5):1084-1099. 10.1158/2159-8290.CD-23-0208 37067240 PMC10164123

[140] Hill W, Lim EL, Weeden CE, et al. Lung adenocarcinoma promotion by air pollutants. *Nature*. 2023;616(7955):159-167. 10.1038/s41586-023-05874-3 37020004 PMC7614604

