# Supplementary material for: Advancing environmental epidemiologic methods to confront the cancer burden
Source: Am J Epidemiol. 2024 Jul 20;194(1):195–207. doi: 10.1093/aje/kwae175 (PMC11735972; doi:10.1093/aje/kwae175)
Supplement: Web_Material_kwae175 [file web_material_kwae175.zip › AJE Supplementary Materials FINAL 7may24.docx]

**Advancing Environmental Epidemiologic Methods to Confront the Cancer Burden**

Rebecca D Kehm, Susan E Lloyd, Kimberly R Burke, Mary Beth Terry

Supplementary Materials:

Table S1. Examples of publicly available national data for environmental linkages

Table S2. Ways that multi-omics platforms can be integrated into epidemiological studies

| Table S1. Examples of publicly available national data for environmental linkages | | | | |
| --- | --- | --- | --- | --- |
| ***Air*** | | | | |
| **Data Source Name** | **Environmental Chemicals** | **Website** | **Years available** | **Geography** |
| CDC Environmental Public Health Tracking Tool | Annual ambient PM 2.5 concentrations;  Daily PM2.5 concentrations over National Ambient Air Quality Standards; Radon | https://ephtracking.cdc.gov/DataExplorer/ | 2000-2018 | Data available at national, state, and county levels |
| EPA Air Quality System | Criteria Air Pollutants  PM2.5, PM10, Ozone, SO2, CO, NO2, VOCs, Hazardous air pollutants | https://www.epa.gov/aqs | 1980- Present | Data are available by monitor, Zip Code, Core-Based Statistical Area (CBSA for metropolitan or micropolitan designation), County Level |
| EPA Ambient Monitoring Archive for HAPs | Hazardous Air Pollutant Emissions | https://www.epa.gov/amtic/amtic-ambient-monitoring-archive-haps | 1990-2020 | Data available at State and Census tract levels |
| National Emission Inventory Data (NEI) | Criteria Pollutant emissions and hazardous air pollutants from all air emissions sources | https://www.epa.gov/air-emissions-inventories/national-emissions-inventory-nei | 2002-present | Zip Code, but state, local, and tribal data are available through the Emissions Inventory System (not publicly available), which is used to build the NEI |
| ***Drinking Water*** | | | | |
| **Data Source Name** | **Environmental Chemicals** | **Website** | **Years available** | **Geography** |
| CDC Environmental Public Health Tracking Tool | Arsenic, Atrazine, DEHP, Disinfection Byproducts, Nitrates, Radium, PCE, PFAS, Perchlorate, TCE, Uranium | https://ephtracking.cdc.gov/DataExplorer/ | 2002-2016 | Available for all 50 states and each state by county |
| EPA Safe Drinking Water Information System | Information about 156,000 public water systems and exceedances of EPA’s drinking water regulations | https://www.epa.gov/enviro/sdwis-overview#:~:text=The%20Safe%20Drinking%20Water%20Information,for%20the%20last%20ten%20years | 2013- Present | Available at the county and city level |
| ***Toxics Data*** | | | | |
| **Data Source Name** | **Environmental Chemicals** | **Website** | **Years available** | **Geography** |
| Toxics Release Inventory (TRI) | Annual toxic chemical releases and waste management activities. There are 770 chemicals in the TRI program. Facilities report on chemical releases that are above established levels. | https://www.epa.gov/toxics-release-inventory-tri-program | 1987- Present | Based on facility; national, ZIP code, state, EPA Region and county level data available through the TRI Explorer |

| Table S2. Ways that multi-omic platforms can be integrated into epidemiological studies | | | | |
| --- | --- | --- | --- | --- |
| **Omic Platform^1,2^** | **Exposure Agent** | **Modifier of the Environmental Agent’s Effect on Cancer** | **Mediator of the Environmental Agent’s Effect on Cancer** | **Intermediate**  **Outcome for Intervention Studies** |
| Germline Genomics | Mendelian Randomization^3,4^ | Yes, but may require enrichment to increase statistical power.^3,4^ | No | No |
| Somatic Genomics | No | Yes, if measured before exposure | Yes, if measured after exposure | Yes, if measured after exposure |
| Epigenomics | No | Yes, if measured before exposure | Yes, if measured after exposure | Yes, if measured after exposure |
| Transcriptomics | No | Yes, if measured before exposure | Yes, if measured after exposure | Yes, if measured after exposure |
| Proteomics | No | Yes, if measured before exposure | Yes, if measured after exposure | Yes, if measured after exposure |
| Metabolomics | No | Yes, if measured before exposure | Yes, if measured after exposure | Yes, if measured after exposure |
| References   1. Hasin Y, Seldin M, Lusis A. Multi-omics approaches to disease. *Genome Biol*. 2017;18(1):83. 2. Karczewski KJ, Snyder MP. Integrative omics for health and disease. *Nat Rev Genet.* 2018;19(5):299-310. 3. Sanderson E, Glymour MM, Holmes MV, et al. Mendelian randomization. *Nat Rev Methods Primers.* 2022;2(1):6. 4. Emdin CA, Khera AV, Kathiresan S. Mendelian Randomization. *JAMA*. 2017;318(19):1925-1926. | | | | |
